# Value of CT perfusion imaging in wake-up strokes – a comparative study of patients qualifying for thrombolysis vs those not suitable for thrombolysis

**DOI:** 10.1016/j.afjem.2026.100988

**Published:** 2026-06-05

**Authors:** Yair Katz, Louis Kroon, Clara-Maria Schutte, Surina Cloete

**Affiliations:** a Steve Biko Academic Hospital, University of Pretoria, South Africa; b University of Pretoria, South Africa

**Keywords:** Advanced neuroimaging, Wake-up stroke, Thrombolysis, Perfusion studies, Stroke risk factors, Emergency

## Abstract

**Background and purpose:**

Stroke remains a major global health concern, contributing significantly to worldwide morbidity and mortality. Despite advances in stroke management, wake-up strokes pose unique challenges for the Emergency Physician due to the uncertain timing of symptom onset, often resulting in limited treatment options. A significant knowledge gap concerning the diagnostic challenges, optimal management and outcomes associated with wake-up strokes remains, particularly in low- and middle-income countries. This study compares outcomes between patients with wake-up strokes who received thrombolysis and those who did not, based on advanced imaging.

**Methods:**

We used a retrospective review of patient records for all wake-up strokes between January 2022 and July 2024 from a Johannesburg, South Africa hospital. Data collected included demographics, risk factors, initial National Institute of Health Stroke Scale and modified Rankin Scale, radiological characteristics, interventions and discharge information. We utilised the RAPID-AI software to aid in analysis of the CT perfusion studies.

**Results:**

Some 57 patients fulfilled the inclusion criteria (51% females), with 22 patients receiving thrombolytic therapy and showed a 65% reduction (median of 12 on admission to 4 on discharge) in median National Institute of Health Stroke Scale score compared to a 45% reduction (median of 11 on admission to 6 on discharge) in those who did not receive thrombolytic therapy (p=0.01). Post-lytic intracerebral haemorrhage without clinical deterioration occurred in one patient. There were 7 mortalities, with 2 in the thrombolytic cohort unrelated to thrombolysis.

**Conclusion:**

In patients presenting with wake-up strokes, computed tomography perfusion can be used successfully to help determine eligibility for thrombolytic therapy. Furthermore, thrombolysed patients had a higher rate of functional recovery.

## African Relevance

• Africa has a disproportionately high burden of strokes, necessitating robust, targeted interventions within the Emergency Care setting.
• By leveraging tissue-based triage through advanced neuroimaging, clinicians can fast-track stroke care.
• This strategy optimises the management of wake-up stroke patients who present to the Emergency Department with an unknown time of symptom onset.
• This research advocates for more equitable access to thrombolysis.

## Introduction

Stroke is a major global health issue, ranking as the second leading cause of death with 7,44 million deaths in 2021 [1] For the survivors of stroke, it may also lead to long-term disabilities that pose significant economic and social challenges. Furthermore, ischaemic stroke incidence is expected to rise globally by 10% by the year 2040 [2]. About 87% of all strokes are ischaemic, and approximately 20–25% have an unknown onset, with the majority of those occurring during sleep and thus classified as "wake-up" strokes [1,3]. In South Africa, it is estimated that between 60 – 80% of all strokes are ischaemic [4,5]. Low- and middle-income countries bear a higher burden, with mortality and disability rates from strokes up to four times higher than in high-income countries, often due to limited access to timely healthcare [2,6]. In South Africa, stroke ranks as the second leading cause of death and is a major contributor to morbidity. Annually, approximately 75,000 individuals experience a stroke in the country, resulting in an estimated 564,000 disability-adjusted life-years (DALY) related to stroke. Furthermore, due to the lack of a national stroke registry and likely under-reporting, these numbers are possibly an underestimate [7].

Significant advances in stroke management began with the approval of tissue plasminogen activator (tPA) in 1996, which shifted the focus to rapidly restoring blood flow [8]. This led to further innovations, such as mechanical thrombectomy, extending the therapeutic window for some patients. While thrombolysis was initially limited to patients with strokes who presented within 4.5 h of onset, the use of advanced imaging, such as computerised tomography (CT) and magnetic resonance imaging (MRI) perfusion studies, has further extended treatment windows. Trials such as the WAKE-UP, DEFUSE-2, and EXTEND studies have shown that, with appropriate imaging, thrombolytic therapy can be effective up to 9 h after symptom onset and mechanical thrombectomy up to 24 h [[9], [10], [11], [12]].

The WAKE-UP trial demonstrated that patients with an unknown time of stroke onset could benefit from intravenous thrombolysis if MRI showed a DWI-FLAIR “mismatch” indicating that the stroke was likely within the 4,5-hour time window [12]. DEFUSE-2 then established the presence of an ischaemic penumbra and thus salvageable tissue identified by perfusion imaging as a better predictor of response to reperfusion therapy than time-based selection. Finally, the EXTEND trial utilised CT perfusion to extend the time-window for intravenous alteplase to 9 h since last known well, if the patients fulfilled the perfusion-based criteria for salvageable tissue [9,10].

While tPA treatment is costly, it has the potential to reduce long-term healthcare expenses by decreasing the need for rehabilitation and allowing patients to return to work [13,14]. In resource-limited settings like South Africa, economic evaluations are essential to justify allocation of funds to stroke care, particularly for wake-up strokes where advanced neuroimaging can identify patients who may benefit from thrombolytic therapy despite the unknown onset time.

Wake-up strokes present a unique challenge to the Emergency Physician due to the uncertain onset, making time-sensitive interventions difficult to apply. In the Emergency Department, these patients were previously approached with a 'therapeutic nihilism' and triaged as 'non-urgent' or excluded from reperfusion pathways. Yet, circadian factors, such as increased stroke risk in the early morning, suggest many wake-up strokes might occur close to waking and thus could be potential candidates for thrombolytic therapy [15,16]. However, even if the stroke occurred earlier in the night, advanced imaging techniques are able to assess tissue viability by demonstrating core and penumbra sizes, thus guiding treatment eligibility [17].

Current guidelines recommend intravenous thrombolysis for wake-up strokes with favourable imaging, provided mechanical thrombectomy is not planned [18]. Unfortunately, in South Africa, access to advanced neuroimaging is limited, restricting treatment options [19]. At Steve Biko Academic Hospital (SBAH), a large tertiary teaching and training hospital, CT perfusion was first introduced in 2022 and this study thus aimed to evaluate outcomes of patients with wake-up strokes treated with thrombolysis based on this imaging modality. This may harbour the potential to advocate for wider access to this approach across South Africa’s public health sector, improving stroke care in the country.

## Methods

### Study population

This retrospective study included all adult patients (>18 years) with wake-up strokes admitted to SBAH between January 2022 and July 2024. Wake-up strokes were defined as ischaemic strokes occurring during sleep (i.e. patient being asymptomatic before sleep and awakening with symptoms). Inclusion criteria followed the American Heart Association/American Stroke Association Expert Consensus definition of stroke [20]. Patients with known stroke onset, unclear onset, wake-up strokes presenting beyond 24 h of last known well and stroke mimics were excluded.

All included patients were admitted to our dedicated acute stroke unit for a minimum of 24 h of specialised care and monitoring prior to transfer to the general neurology ward. Management protocols, including blood pressure targets, nursing care, and early rehabilitation, were identical for both the thrombolysis and conservative cohorts to ensure that outcomes were reflective of the reperfusion intervention rather than variations in supportive care. For the thrombolysis group, follow-up neuroimaging was performed within 24 h of treatment to assess for haemorrhagic transformation. While patients with large vessel occlusions were identified, mechanical thrombectomy was not performed in any cases due to resource limitations at our centre.

### Data collection

Consecutive sampling was used to ensure all eligible cases were included, with data collected from the hospital's Registry of Stroke Care Quality (RES-Q) Health Data Management Platform and cross verified with the department’s stroke database to avoid selection bias. We collected clinical and demographic data including age, sex, comorbidities, social habits, time of last known well, timeframes relevant to the care process and biochemical markers. Clinical stroke severity was determined using the National Institute of Health Stroke Scale (NIHSS) and Modified Rankin Scale (mRS) scores at admission and discharge.

Neuroimaging characteristics, including Alberta Stroke Programme Early CT Score (ASPECTS), vascular involvement, and perfusion metrics were used to guide eligibility for thrombolysis based on core infarct size (< 70 ml), penumbra volume (> 10 ml), and mismatch ratio (> 1.2) with the help of RAPID-AI. Information on treatment, such as thrombolysis administration, post-treatment imaging and clinical improvement was reviewed. We utilise RAPID AI software to evaluate thrombolysis eligibility based on the European Stroke Organisation (ESO) Stroke Guidelines, which align with the EXTEND-IA trial criteria [9,18].

### Statistics

The statistical analysis plan, outlined in the study protocol, was finalised before the database was locked. Analyses were conducted using Stata software, version 18 (StataCorp). The sample size was calculated based on THRACE III outcomes, which estimated that 33% of patients would meet the criteria for reperfusion using advanced neuroimaging.

Statistical significance was defined as a two-sided p-value of <0.05. The distribution of continuous variables was assessed using the Shapiro Wilk test and visual inspection of histograms. Given the presence of non-normal data, non-parametric tests were used. The Mann-Whitney U test (Wilcoxon rank-sum) was applied for comparisons between two groups, and the Kruskal Wallis test for comparisons across multiple groups. Categorical variables were analysed using the chi-square test or Fisher’s exact test where appropriate. Correlations between continuous variables were assessed using Spearman’s rank correlation coefficient.

### Ethical approval

This study was approved by the University of Pretoria Faculty of Health Sciences Research Ethics Committee which is accredited nationally by the National Health Research Ethics Council of the South African Department of Health as well as internationally by the Office of Human Research Protection of the USA Department of Health and Human Services (Reference Number: 409/2024).

This manuscript conforms to the STROBE (Strengthening the Reporting of Observational Studies in Epidemiology) reporting guideline and checklist.

## Results

Between January 2022 and July 2024, 513 ischaemic stroke patients were admitted to the SBAH stroke unit, with 57 confirmed wake-up strokes (11,1%). There were 29 females with a mean age of 68 years (range 31 – 87 years) compared to 28 males with a lower mean age of 55 years (range 37 – 73 years). Baseline characteristics of the patients are shown in Table 1.

**Table 1:** Patient characteristics and outcomes.

Characteristic	Thrombolysis (n = 22)	Conservative (n = 35)
Mean Age (range):	Males: 54 years (42 – 68) Females: 68 years (31 – 87)	Males: 56 (37 – 73) Females: 67 (36 – 84)
Thrombolysis:	Males: 11 Females: 11	Males: 18 Females: 17
Mean Aspect Score (range):	8,87 (7 – 10)	6,96 (0 – 10)
CT Perfusion: * Average Core Size (range)	20,3 ml (0 – 63 ml)	38,8 ml (0 – 144 ml)
CT Perfusion: Average Penumbra Size (range)	69,1 ml (0 – 204 ml)	30,3 ml (0 – 206 ml)
Median NIHSS on Admission:*	12 (IQR: 8 – 16)	11 (IQR: 6 – 16)
Median NIHSS on Discharge:	4 (IQR: 2 – 7)	6 (IQR: 4 – 14)
Median mRS on Admission:*	4 (IQR: 3 – 5)	4 IQR: 3 – 5)
Median mRS on Discharge:	1 (IQR: 1 – 3)	2 (IQR: 2 – 4)
Median time from LKW to Presentation (minutes)*	780 (IQR: 622 – 936)	600 (IQR: 510 – 717)
Median Door to Imaging Time (minutes)	15,5 (IQR: 13 – 25)	17 (IQR: 11 – 33)
Door to Needle Time (minutes)	29 (IQR: 20 – 35)	N/A
Length of Admission (days)	8 (IQR: 7 – 10)	10 (IQR: 4 – 15)

*CT, Computed Tomography; NIHSS, National Institute of Health Stroke Scale; mRS, Modified Rankin Scale; LKW, Last Known Well; IQR, Interquartile Range.

CT perfusion studies were performed in 49/57 patients (86%), with 22 patients (39%) identified as having salvageable tissue to receive intravenous alteplase.

Efficiency of care within the Emergency Department was high, with a median door-to-imaging time of 15.5 min (IQR: 13–25) and a door-to-needle time of 29 min (IQR: 20–35) for the thrombolysis cohort. These intervals reflect the feasibility of rapid hyperacute stroke protocols in a busy public-sector Emergency Department.

**Fig. 1:**
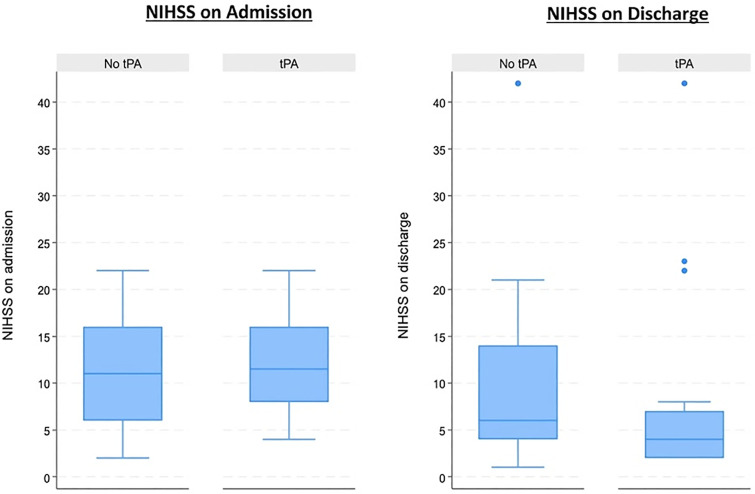
a: NIHSS distribution on admission by lytic group. b: NIHSS distribution on discharge by lytic group * tPA, Tissue Plasminogen Activator; NIHSS, National Institute of Health Stroke Scale.

As illustrated in Fig. 1a, patients who received thrombolysis had a median NIHSS score of 12 (IQR: 8–16) upon presentation to the emergency department, while those who did not receive thrombolysis had a median score of 11 (IQR: 6–16). By the time of discharge, the thrombolysis group’s NIHSS scores reduced by 65%, with a new median score of 4 (IQR: 2–7). In contrast, the non-thrombolysis group’s scores decreased by 45%, with a median score of 6 (IQR: 4–14; Fig. 1b). This 20% difference between the two groups showed a statistically significant difference (p = 0.01).

**Fig. 2:**
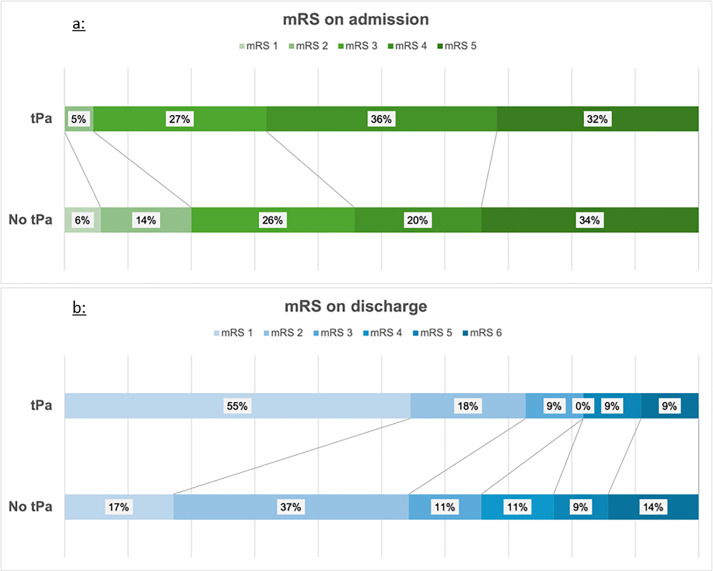
a: mRS on admission in thrombolytic group and conservative group b: mRS at discharge in thrombolytic group and conservative group * tPA, Tissue Plasminogen Activator; mRS, Modified Rankin Scale.

Initial assessment within the Emergency Department demonstrated that 68% of patients who subsequently received thrombolysis presented with a modified Rankin Scale (mRS) score of 4 or more, compared with 54% of the non-thrombolysis cohort (Fig. 2a). By the time of hospital discharge, 55% of patients treated with thrombolysis achieved a favourable functional outcome (mRS 0–1), contrasted with only 17% of those in the non-thrombolysis group (Fig. 2b). Furthermore, clinical improvement was observed in 77% of the thrombolysed cohort compared to 51% of non-thrombolysed patients. Conversely, a smaller proportion of the thrombolysis group (9%) experienced clinical worsening, compared to 17% in the non-thrombolysis cohort.

Using a two-sample Wilcoxon rank-sum (Mann-Whitney) test to compare the NIHSS change between both groups (thrombolysis vs conservative) for each categorisation within the Oxfordshire classification, there was a statistically significant difference in NIHSS change between the lytic and non-lytic groups for anterior circulation strokes (z-vale of -2407; p-value 0.015). For posterior circulation strokes (p-value 0.4), lacunar infarcts (p-value 0.4) and multi-vascular infarcts (p-value 0.5), no clear difference between the groups was found.

The CT scans were assessed for the presence or absence of previous infarcts and lacunar infarcts. The Wilcoxon rank-sum (Mann-Whitney) test revealed a p-value of 0.331, indicating that there was no statistically significant difference in the NIHSS change distributions between patients with previous infarcts and those without. Similarly, lacunar infarcts did not show a significant difference, with a p-value of 0.857. Therefore, neither the presence nor the type of old infarct significantly affected the change in NIHSS scores. Interestingly, 46 patients (81%) demonstrated evidence of small vessel disease on neuroimaging, with lacunar infarcts seen in 56% of cases, either alone or in combination with leukoaraiosis in 71% of cases.

In-hospital mortality was 12% (7 deaths). Of these, 2 were in the thrombolysis group but neither death was related to thrombolytic therapy. All 7 patients who died, had presented with proximal middle cerebral artery (M1) large vessel occlusions, with a mean NIHSS of 16 and ASPECT scores of 5 and 6 upon arrival.

Using a Spearman Rank Correlation test, there was a moderate statistically significant negative correlation (Spearman’s rho -0.416) between NIHSS score and ASPECT score (p-value: 0.0014) and a moderate negative correlation (Spearman’s rho -0.5459) between the NIHSS discharge score and the ASPECT score (p-value: 0.0001).

Regarding complications, one patient had a post-lytic intracranial haemorrhage, confined to the infarct with no mass effect (haemorrhagic infarction type-1b; HI-1b) and no clinical deterioration. Additionally, the patient improved clinically with discharge mRS and NIHSS scores improving to 1 and 2 from admission levels of 5 and 11, respectively.

## Discussion

Advanced neuroimaging has transformed the management of wake-up strokes by providing visualisation into the ischaemic penumbra and the realisation that ischaemic stroke progression may vary among patients. Some experience slow progression, preserving penumbral tissue and thus potentially benefiting from delayed reperfusion therapy, while others quickly lose salvageable tissue. The "tissue clock" concept highlights individual variability in ischaemic progression over time. Thus, advanced neuroimaging is critical to allow for better assessment of tissue viability and perfusion deficits, enabling tailored treatment for this previously overlooked group [9,[21], [22], [23]]. Our findings clearly show that selected patients with wake-up strokes may benefit from thrombolytic therapy in our setting. Almost half of the patients who had perfusion CT studies qualified for thrombolysis. This group had a significantly better outcome compared to patients with wake-up strokes who had large infarct core volumes or penumbra <10 ml who were not candidates for thrombolytic therapy. A greater NIHSS reduction at discharge (65%) in the thrombolysis treated group vs the non-thrombolysis group (45%; p-value 0.01) confirms that there is a benefit to actively treat these patients. Functional recovery as measured by the mRS and compared between the two groups (mRS 0–1: 55% vs. 17%) also showed significant improvement in the thrombolysis treated patients. In a 2018 study by Caruso et al., the efficacy of CT perfusion for thrombolysis in wake-up strokes was assessed. Despite some differences in the perfusion criteria compared to ours, thrombolysis showed a statistically significant improvement in NIHSS and mRS scores, similar to those treated within 4.5 h from symptom onset. The researchers concluded that neuroimaging-guided decision making for thrombolytic therapy based on salvageable penumbra may extend the time window by tailoring treatment to patients in order to maximise the potential benefit and minimise the risks, a finding supported by our investigation [24]. In 2019, the EXTEND trial, also employing perfusion imaging with the RAPID-AI software and the same thrombolytic eligibility criteria that we used, showed a higher percentage of patients with no/minor neurological deficits in the thrombolysis group compared to placebo in patients presenting with strokes between 4.5 and 9 h and including wake-up strokes. Overall, 35% of patients who had received thrombolysis achieved good functional outcomes (mRS 0–1) at 90 days. This trial was discontinued early when the positive results of the WAKE-UP trial were published [10]. In the meta-analysis regarding intravenous alteplase for stroke of unknown onset guided by advanced imaging, Thomalla et al. showed that there was a 47% favourable outcome (mRS 0–1) for thrombolysis-treated patients which is comparable to our finding of 55% [11].

The overall mortality (12%) in our group of patients was linked to severe strokes as noted by high NIHSS (mean of 16 on arrival), low ASPECT scores and large vessel occlusions. Mortality in the thrombolysis group was 9% and only one patient had an asymptomatic post-lytic haemorrhage. In the EXTEND trial, mortality was 11.5% in the alteplase arm and 6.2% of the patients had haemorrhages, all of them reported as symptomatic (of whom 2 died) [10]. Intracranial haemorrhage occurred in 23% of patients and caused symptomatic deterioration in 9% of patients with wake-up strokes in Caruso et al’s study, while the meta-analysis by Thomalla (which included EXTEND) showed 3% of symptomatic haemorrhages post thrombolysis with a 6% mortality – comparing well with our findings [11,24]. Strict adherence to the ESO Stroke Guidelines for determining suitability of patients for thrombolysis with advanced neuroimaging appears to minimise post-thrombolytic haemorrhages in wake-up strokes.

Our study cohort demonstrated a high vascular burden, with hypertension (65%) and dyslipidaemia (63%) notably more prevalent than in global meta-analyses. Despite 70% of patients possessing two or more risk factors and 56% exhibiting radiographic evidence of small vessel disease, a significant diagnostic gap exists; many patients were unaware of their conditions prior to admission, underscoring a critical need for improved primary care screening in South Africa.

Mechanical thrombectomy as a successful treatment modality for ischaemic strokes with large vessel occlusions has been proven in the AURORA collaboration with 45,9% of patients achieving no or mild disability in the intervention arm compared to 19,3% in the those receiving best medical therapy [[25], [26], [27]]. However, in low- and middle-income countries (LMIC) there are significant challenges due to the shortage of skilled interventionalists and interventional suites, coupled with the high cost of equipment [19,28]. Nevertheless, to establish an effective, nationally coordinated stroke service, it is imperative to prioritise the training of clinicians and formation of dedicated stroke teams within hospitals, as well as the development of regional networks to facilitate the rapid transfer of suitable patients to comprehensive stroke centres [19]. The first step to successfully establishing this network is to increase the availability of advanced neuroimaging.

Our study is limited by only describing the experience of a single-centre as well as the lack of blinded outcome assessments for NIHSS and mRS scores that may have introduced assessment bias. Furthermore, our small sample size reduces the statistical power of the study and the absence of mechanical thrombectomy capabilities restricted the management of large vessel occlusions to medical therapy alone. However, despite being a retrospective study, consecutive sampling was used to prevent selection bias.

## Conclusion

This study highlights the ability in a public healthcare centre in a LMIC to successfully treat wake-up strokes – a previously neglected group of patients, showing similar positive outcomes to those seen in high-income countries. Additionally, it emphasises the critical role of advanced neuro-imaging in determining eligibility for and safety of thrombolytic therapy and prioritising tissue-based criteria over conventional clock-time based assessments. Furthermore, this study empowers Emergency Medicine Physicians and advocates for similar wake-up stroke protocols to be replicated in other Emergency Departments in South Africa and elsewhere in order to enable better outcomes for these patients. In a limited resource setting, we have shown that without timeous access to MR imaging and thrombectomy capabilities, we can still have an impactful role in successfully managing this important subset of stroke patients.

Future research should prioritise identifying and mitigating systemic barriers to implementing advanced neuroimaging within the South African public healthcare sector. Furthermore, incorporating mechanical thrombectomy for wake-up stroke patients with large vessel occlusions should be explored to establish context-specific evidence. Multicentre collaborative trials within the public healthcare setting are suggested with the goal of optimising stroke care efficiencies and ensuring that life-saving reperfusion therapies are both accessible and sustainable within the South African socioeconomic landscape.

## Dissemination of results

The findings from my research were shared at the Neurology Association of South Africa Congress and at the University of Pretoria Faculty Research Day. It is our intention to make these findings available to policymakers and fellow academics through a publication in the African Journal of Emergency Medicine.

## Data availability statement

Data will be uploaded and made available on OSF.io for transparency and availability on request.

## CRediT authorship contribution statement

**Yair Katz:** Conceptualization, Data curation, Investigation, Methodology, Project administration, Writing – original draft, Writing – review & editing. **Louis Kroon:** Conceptualization, Methodology, Supervision, Writing – review & editing. **Clara-Maria Schutte:** Conceptualization, Investigation, Supervision, Formal analysis, Validation, Writing – review & editing. **Surina Cloete:** Formal analysis, Software, Validation.

## Declaration of conflicting interests

The authors declared no potential conflicts of interests.
